# Supplementary figures and images for: The Characteristics of 206 Long-Term Survivors with Peritoneal Metastases from Colorectal Cancer Treated with Curative Intent Surgery: A Multi-Center Cohort from PSOGI
Source: Cancers (Basel). 2021 Jun 13;13(12):2964. doi: 10.3390/cancers13122964 (PMC8231850; doi:10.3390/cancers13122964)

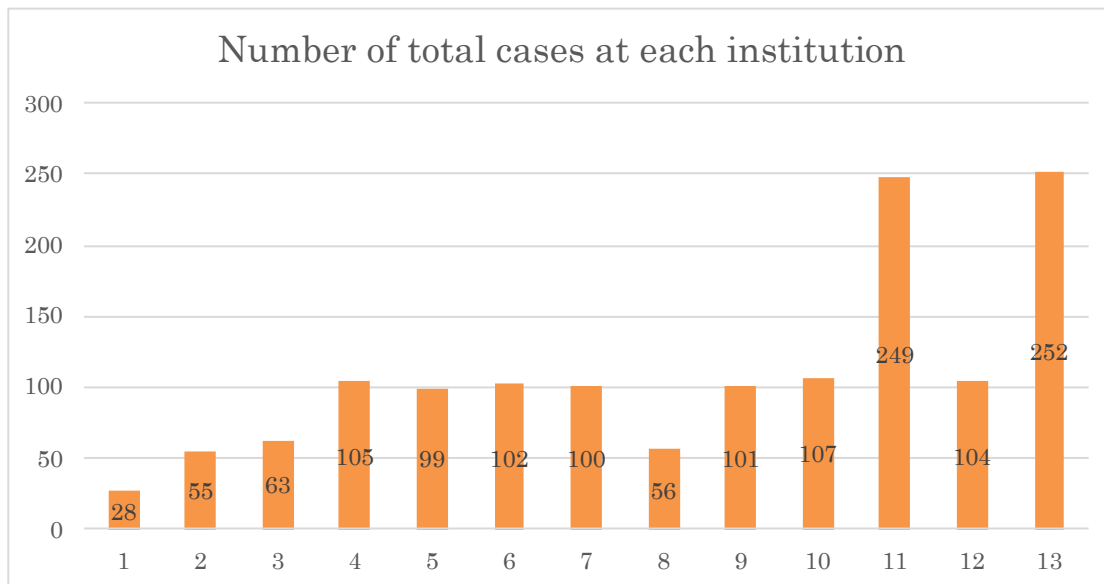

Figure S1

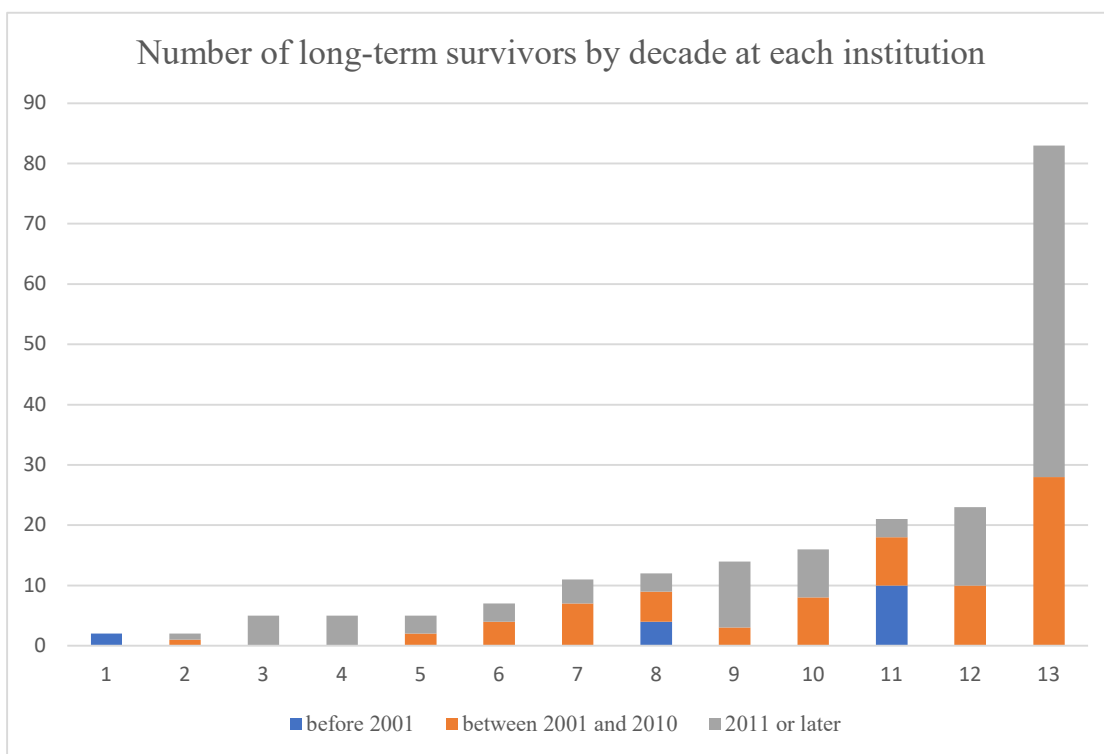

Figure S2

Supplement: Supplementary file 1 [file cancers-13-02964-s001.zip › cancers-1244472-supplementary.pdf]
